# Toll-Like Receptor 4 Upregulation by Angiotensin II Contributes to Hypertension and Vascular Dysfunction through Reactive Oxygen Species Production

**DOI:** 10.1371/journal.pone.0104020

**Published:** 2014-08-05

**Authors:** Priscila R. De Batista, Roberto Palacios, Angela Martín, Raquel Hernanz, Cindy T. Médici, Marito A. S. C. Silva, Emilly M. Rossi, Andrea Aguado, Dalton V. Vassallo, Mercedes Salaices, María J. Alonso

**Affiliations:** 1 Dept. of Biochemistry, Physiology and Molecular Genetics, Universidad Rey Juan Carlos, Alcorcón, Spain; 2 Dept. of Physiological Sciences, Federal University of Espirito Santo, Vitoria, Brazil; 3 Dept. of Pharmacology, Universidad Autónoma de Madrid, Madrid, Spain; The Chinese University of Hong Kong, Hong Kong

## Abstract

Hypertension is considered as a low-grade inflammatory disease, with adaptive immunity being an important mediator of this pathology. TLR4 may have a role in the development of several cardiovascular diseases; however, little is known about its participation in hypertension. We aimed to investigate whether TLR4 activation due to increased activity of the renin-angiotensin system (RAS) contributes to hypertension and its associated endothelial dysfunction. For this, we used aortic segments from Wistar rats treated with a non-specific IgG (1 µg/day) and SHRs treated with losartan (15 mg/kg·day), the non-specific IgG or the neutralizing antibody anti-TLR4 (1 µg/day), as well as cultured vascular smooth muscle cells (VSMC) from Wistar and SHRs. TLR4 mRNA levels were greater in the VSMC and aortas from SHRs compared with Wistar rats; losartan treatment reduced those levels in the SHRs. Treatment of the SHRs with the anti-TLR4 antibody: 1) reduced the increased blood pressure, heart rate and phenylephrine-induced contraction while it improved the impaired acetylcholine-induced relaxation; 2) increased the potentiation of phenylephrine contraction after endothelium removal; and 3) abolished the inhibitory effects of tiron, apocynin and catalase on the phenylephrine-induced response as well as its enhancing effect of acetylcholine-induced relaxation. In SHR VSMCs, angiotensin II increased TLR4 mRNA levels, and losartan reduced that increase. CLI-095, a TLR4 inhibitor, mitigated the increases in NAD(P)H oxidase activity, superoxide anion production, migration and proliferation that were induced by angiotensin II. In conclusion, TLR4 pathway activation due to increased RAS activity is involved in hypertension, and by inducing oxidative stress, this pathway contributes to the endothelial dysfunction associated with this pathology. These results suggest that TLR4 and innate immunity may play a role in hypertension and its associated end-organ damage.

## Introduction

Hypertension has been generally associated with structural and functional vascular alterations, and both endothelial dysfunction and increased vasoconstrictor responses are important features of this pathology. The reduced nitric oxide (NO) bioavailability caused by increased reactive oxygen species (ROS) production would explain these vascular alterations. In this context, in recent years, it has been proposed that low-grade inflammation plays a key role in the development and progression of hypertension [1]–[4]. Indeed, in hypertension, increases in the plasma levels of proinflammatory cytokines [1], in the ROS production [5], [6] and in the vascular responses to lipopolysaccharide (LPS) [7]–[9] have been observed. It is worth noting that inflammation also induces endothelial dysfunction in humans and animals [9], [10]. Increased activation of the renin-angiotensin system (RAS) seems to be associated with the inflammatory state observed in hypertension, as well as with its associated vascular alterations [1], [5], [11], [12]. Angiotensin II (Ang II), the effector peptide of RAS, is able to induce Toll-like Receptor 4 (TLR4), and it seems that TLR4-dependent signaling pathway contributes to the proinflammatory effects of this humoral factor [13]–[19].

TLRs belong to a large family of pattern recognition receptors that play important roles in mammalian defense systems against invading microorganisms. Among then, TLR4 is expressed on the surface of several cell types, including endothelial and vascular smooth muscle cells (VSMCs). It recognizes and responds against LPS, the main component of the cell wall of Gram-negative bacteria, as well as other non-infectious compounds, such as the products of tissue death and/or damage (DAMP), heat shock proteins (Hsp), high-mobility group box 1 (HMGB1) protein, fibronectin, heparan sulfate and fibrinogen. After activation, TLR4 can initiate the innate and, subsequently, the adaptive immunity; both mechanisms are responsible for the inflammatory response [20], [21]. It has also been shown that upon TLR4 activation, LPS produces ROS such as superoxide anion and hydrogen peroxide [22]–[25], which also contribute to the inflammatory response.

The roles of the TLR4 signaling pathway in the processes underlying inflammatory vascular diseases including atherosclerosis [14], diabetes [26]–[28] or pre-eclampsia [29], [30] have been reported. While several studies have addressed the contribution of adaptive immunity to the pathophysiology of hypertension, there are few studies regarding the role of the innate immune system in the context of this pathology [31]–[33]. Therefore, the aim of this study was to investigate whether TLR4 activation, due to increased RAS activity, contributes to hypertension and the functional vascular alterations observed in this pathology. The specific objectives were to investigate the following: 1) the alteration of TLR4 expression in hypertension and the contribution of Ang II to this alteration; 2) the role of TLR4 in hypertension occurrence, as well as in the associated vascular function alterations; and 3) the involvement of the TLR4-activated ROS production in the vascular dysfunction associated to this pathology.

## Materials and Methods

### Ethics statement and Animals

All experiments were approved by the Ethical Commission for the Use of Animals of Universidade Federal do Espírito Santo, Brazil (CEUA-UFES 042/2013) and by the Ethical Committee of Research of the Universidad Autónoma de Madrid, Spain (CEI-UAM 31-759). This study was carried out in strict accordance with the recommendations for biomedical research as stated by the Brazilian Societies of Experimental Biology, the guidelines for ethical care of experimental animals of the European Community, the current Spanish and European laws (RD 223/88 MAPA and 609/86), and the International Guiding Principles for Biomedical Research Involving Animals.

Adults male spontaneously hypertensive (SHRs) and Wistar rats were used for these studies. Rats were housed under a 12 h light/12 h dark cycle, they had free access to water and were fed a standard rat chow *ad libitum*. In one set of experiments, we analyzed if hypertension alters TLR4 expression and its dependence on RAS activity. For this, we used Wistar rats and SHRs untreated and treated with the AT_1_ receptor antagonist losartan (15 mg/kg·day in the drinking water, 12 weeks; generously supplied by Merck & Co., Inc., Rahway, NJ, USA). Systolic arterial pressure was measured by tail plethysmography.

In another set of experiments, we investigated whether the TLR4 receptor plays a role in the occurrence of hypertension and the associated vascular alterations. For this, we used SHRs (258.5±10.9 g, n = 10) treated with an anti-TLR4 antibody (rat monoclonal IgG_2a_, 1 µg/day saline-diluted, intraperitoneal injection, 15 days; sc-13591, Santa Cruz Biotechnology Inc., Dallas, TX, USA) and Wistar rats (272.1±15.7 g, n = 9) and SHRs (245.9±12.5 g, n = 9) treated with a non-specific IgG (IgG_2a_, 1 µg/day, saline-diluted, intraperitoneal injection, 15 days; sc-2026, Santa Cruz Biotechnology Inc.) to rule out non-specific effects of the anti-TLR4 antibody treatment [32]. Hemodynamic parameters and vascular function in aortic rings were evaluated. To further elucidate the role of TLR4 in the Ang II effects, cell culture experiments using VSMCs from Wistar and SHR were used.

Rats were euthanized by CO_2_, and all efforts were made to minimize suffering. Then, the aortas were removed and placed in cold (4°C) Krebs-Henseleit solution (KHS) (115 mM NaCl, 25 mM NaHCO_3_, 4.7 mM KCl, 1.2 mM MgSO_4_.7H_2_O, 2.5 mM CaCl_2_, 1.2 mM KH_2_PO_4_, 11.1 mM glucose, and 0.01 mM Na_2_EDTA) aerated with a 95% O_2_-5% CO_2_ mixture (pH = 7.4). Aortic segments were dissected free of fat and connective tissue and maintained in KHS. Segments used for gene expression studies were immediately frozen in liquid nitrogen and kept at −70°C until the day of the experiment. The hearts were removed to assess cardiac hypertrophy. For this, the ratio between the heart dry weight and the length of the tibia was calculated.

### Hemodynamic parameters

At the end of the treatment, body weight was recorded and the rats were anesthetized with urethane (4 g/kg of body weight, intraperitoneal injections). The right coronary artery was cannulated with a heparinized polyethylene catheter (PE-50) and connected to a data acquisition system with pressure transducers (TSD 104A, Biopac Systems, Inc., Goleta, CA, USA) to measure hemodynamic parameters. Following an adaptation period of 30 min, systolic, diastolic, mean blood pressure and heart rate (SBP, DBP, MBP and HR, respectively) measurements were recorded.

### Vascular function

Vascular function was studied in aortic segments by isometric tension recording using an isometric force transducer (TSD 125C) connected to an acquisition system (Biopac Systems, Inc.). Segments were initially exposed to 75 mM KCl to test their functional integrity, and the presence of endothelium was confirmed by the effect of 10 µM acetylcholine in segments that previously contracted with 1 µM phenylephrine. After a washout period, a single concentration-response curve to phenylephrine (0.1 nM–0.3 mM) or acetylcholine (0.01 nM–0.3 mM) was performed. Thus, parallel experiments in different aortic segments from the same animal were performed in the absence (control) and the presence of the NAD(P)H oxidase inhibitor apocynin (30 µM), the superoxide anion scavenger 4, 5-dihydroxy-1, 3-benzene-disulphonic acid (tiron, 1 µM) and the hydrogen peroxide detoxificant catalase (1000 U/ml). These drugs were administered 30 min prior to incubation with phenylephrine or acetylcholine.

The influence of endothelium on the response to phenylephrine was investigated after mechanical removal of this vascular component by rubbing the lumen with a needle. The absence of endothelium was confirmed by the inability of 10 µM acetylcholine to produce relaxation. To evaluate the NO component of the phenylephrine responses, the aortic rings were half-maximally pre-contracted with 1 µM phenylephrine for 30 min; then, a non-selective inhibitor of NO synthesis, N^G^-nitro-L-arginine methyl ester (L-NAME, 100 µM), was added for 45 min. The results of the additional tone caused by L-NAME were expressed as the % of the previous contraction elicited by phenylephrine [34].

### Immunofluorescence

TLR4 was immunolocalized as described [12]. Briefly, frozen transverse sections (14 µm) were cut on to gelatin coated slides and air-dried for at least 60 min. After blockade, sections were incubated with a polyclonal antibody against TLR4 (1∶100, Santa Cruz Biotechnology, Inc.) in PBS containing 2% bovine serum albumin (BSA, Sigma Chemical Co.) for 1 h at 37°C in a humidified chamber. After washing, rings were incubated with the secondary antibody, a goat anti-rat (1∶200) IgG labeled with alexa fluor-546 dye (Molecular Probes Inc., Eugene, OR, USA) for 1 h at 37°C in a humid box. After washing, immunofluorescent signals were viewed using an inverted Leica TCS SP2 confocal laser-scanning microscope with oil immersion lens (x40). Alexa Fluor-labeled antibody was visualized by excitation at 546 nm and detection at 550–650 nm. The specificity of the immunostaining was evaluated by omission of the primary antibody and processed as above. Under these conditions, no staining was observed in the vessel wall. Nuclei were stained with 0.01 mg/ml DAPI (Molecular Probes Life Technologies) and visualized with excitation/emission wavelengths of 358/461 nm.

### Cell Culture

To obtain primary cultures of VSMCs, thoracic aortas from SHRs or Wistar rats were aseptically removed, cleaned of fat tissue and blood cells and placed in cold Dulbecco’s modified Eagle’s medium (DMEM) (Sigma Chemical Co., St. Louis, Mo, USA) containing 0.1% BSA, 200 U/ml penicillin and 200 µg/ml streptomycin. The aortas were digested in the same medium containing 2 mg/ml collagenase type II (Worthington, Lakewood, NJ, USA) and incubated for 30 min at 37°C in a humidified atmosphere of CO_2_ (5%). After the adventitia was carefully removed, VSMCs were obtained using the explant method [35]. Cells were identified as VSMCs by morphological and growth characteristics and by positive immunocytochemical staining with a specific monoclonal anti-α-actin antibody (Sigma Chemical Co.). For experiments, cells from passages 2 to 5 were rendered quiescent by incubation in DMEM containing 0.2% FBS for 24 h. The cells were stimulated with 100 nM Ang II (for the times indicated in the results section), with or without pretreatment for 1 h with the TLR4 inhibitor CLI-095 (1 µM). The specificity of CLI-095 was confirmed by its capacity to abolish the induction of COX-2 expression in VSMCs following exposure to LPS (data not shown).

### Quantitative PCR real time (qRT-PCR) assay

TLR4, NOX-1, NOX-2, NOX-4 and p22phox mRNA levels were determined in the aortic segments and/or VSMCs by qRT-PCR. Total RNA was obtained using the TRI Reagent (Sigma Chemical Co.), according to the manufacturer’s recommendations, and was reverse-transcribed using the High Capacity cDNA Archive Kit (Applied Biosystems, Foster City, CA, USA). PCR was performed using the fluorescent dye SyBR Green (iTaq FAST SyBR Green Supermix with ROX, Bio-Rad Laboratories, Hercules, CA, USA) or using Taqman Gene Expression Assays (NOX-1: Rn00586652_m1; NOX-4: Rn00585380_m1, Applied Biosystems). The primer sequences used are: TLR4 (FW: TGTGCCTTCAAAACATGACTGG, RV: CTCCCAAGATCAACCGATGG); p22phox (FW: GGACAGAAGTACCTGACCGC, RV: GATGGTGGCCAGCAGGAAG); NOX-2 (FW: CCAGTGAAGATGTGTTCAGCT, RV: GCACAGCCAGTAGAAGTAGAT). Cyclophilin D (Rn01458749_g1, Applied Biosystems) and β2-microglobulin (Rn00560865_m1, Applied Biosystems) were used as normalizing internal controls. All PCRs were performed in duplicate. qRT-PCR was carried out in an ABI PRISM 7000 Sequence Detection System (Applied Biosystems, from the Centro de Apoyo Tecnológico of URJC) using the following conditions: 2 min at 50°C; 10 min at 95°C; and 40 cycles of 15 s at 95°C and 1 min at 60°C. At the end of the SyBR Green PCR, a final stage with a melting curve analysis was added to show the specificity of the product. To calculate the relative index of gene expression, we employed the 2^−ΔΔCt^ method using the untreated samples for calibration.

### Detection of ROS

The oxidative fluorescent dye dihydroethidium (DHE) was used to evaluate *in situ* superoxide anion production in VSMCs [35]. Hydroethidine freely permeates cells, and in the presence of superoxide anions it is oxidized to ethidium bromide, which is trapped by intercalation with DNA. Ethidium bromide is excited at 546 nm and has an emission wavelength of 610 nm. Briefly, VSMCs were plated onto glass coverslips placed in 6-well plates and cultured as described above. Subconfluent cells were stimulated with 100 nM Ang II for 2 h in the absence or presence of 1 µM CLI-095, which was added 1 h prior Ang II. The cells were then incubated with 10 µM DHE in cell culture medium for 30 min at 37°C. The images were then acquired using a fluorescence microscope (Nikon Eclipse T300, objective ×20, Nikon Corporation, Tokyo, Japan), captured using a digital spot camera (Diagnostic, Spectra Services, Ontario, NY, USA) and processed using the Metamorph image analysis software (Molecular Devices Corp., Downingtown, PA, USA). Non-stimulated VSMCs were imaged daily in parallel, using the same image settings throughout the course of the study. DHE fluorescence was quantified in individual cell nuclei (10–20 nuclei/image/experimental condition). At least 5 independent experiments were performed. Then, we expressed the effects of the different drugs as fold increases over the control.

### NAD(P)H oxidase activity

The superoxide anion generated by NAD(P)H oxidase activity was determined using a chemiluminescence assay using 5 µM lucigenin and 100 µM NAD(P)H. VSMCs treated as described for the ROS detection experiments were homogenized in a lysis buffer (50 mM KH_2_PO_4_, 1 mM ethylene glycol tetraacetic acid, 150 mM sucrose, pH = 7.4). The reaction was initiated with the addition of a mixture of lucigenin and NAD(P)H to the protein sample in a final volume of 300 µl. Chemiluminescence was determined every 2.4 s for 5 min in a plate luminometer (Auto-Lumat LB 953, Berthold Technologies GmbH & Co. KG, Bad Wildbad, Germany). A buffer blank was subtracted from each reading, and the value of the area under the curve was used to quantify chemiluminescence. The data obtained (counts/s) were expressed as fold increases over the control situation.

### *In vitro* wound healing assay

To verify the role of TLR4 in Ang II-induced migration, a wound healing assay was performed. For this, the cells were seeded and cultured to confluence in a 24-well plate. Then, the cells were switched to serum-free medium for 24 h before the initiation of the experiments. A wound was made with a P10 pipette tip (CRP, with a filter). The medium was changed twice (5 ml/well) to wash away any cell debris remaining in the wound area. A line was drawn through the center of the wells, perpendicular to the wound. A picture was taken at time zero at the site of intersection of the line and the wound. Then, the cells were treated for 1 h with 1 µM CLI-095, followed by 100 nM Ang II. At 24 h, we took a picture in the same location. Adobe Photoshop CS2 was used to determine the area of wound closure compared to time 0 for the stimulus and with respect to the control situation.

### Cell proliferation assay

Cell proliferation was assessed using the CellTiter 96 Non-Radioactive Cell Proliferation Assay (Promega Corporation, Madison, WI, USA). For this, VSMCs were seeded in 96-well plates (20×10^3^ cells/well) in DMEM and were allowed to attach for 24–36 h. Then, the cells were switched to serum-free medium for 24 h. After this, cells were treated with 1 µM CLI-095 for 1 h, followed by 100 nM Ang II for 24 h. The proliferative response was quantified by adding MTS tetrazolium solution (20 µl/well). After 2–3 h of incubation, absorbance was measured at 490 nm using a microplate reader (ASYS Hitech GmbH, Eugendorf, Austria). Different assays were performed in triplicate.

### Drugs and reagents

l-phenylephrine hydrochloride, acetylcholine chloride, tiron, catalase, apocynin, lucigenin, salts and other reagents were purchased from Sigma Chemical Co. and Merck (Darmstadt, Germany). DHE, streptomycin and penicillin were obtained from Invitrogen (Carslbad, CA, USA), and CLI-095 was obtained from Invivogen (San Diego, CA, USA). All drugs were dissolved in distilled water, except for CLI-095, which was dissolved in dimethyl sulfoxide (DMSO); 10 µl of DMSO did not have any effect on VSMCs.

### Statistical analyses

Vasoconstrictor responses induced by phenylephrine were expressed as the % of the tone generated by 75 mM KCl. Vasodilator responses induced by acetylcholine were expressed as the % of the previous tone in each case. The maximum response (Emax) and pD_2_ values were calculated by non-linear regression analysis of each individual concentration-response curve using GraphPad Prism Software. To compare the effect of endothelium removal on the response to phenylephrine in segments from the three groups, the results are expressed as the differences of areas under the concentration-response curves (dAUC) in the control and experimental situations. AUCs were calculated from the individual concentration-response curve plots using a computer program (GraphPad Prism Software, San Diego, CA, USA); the differences were expressed as the % of the AUC of the corresponding control situation.

The results are expressed as the mean±SEM (standard error of the mean) of the number of animals or the number of different cultures used in each experiment; differences were analyzed using Students *t*-test or one- or two-way analyses of variance (ANOVA), followed by the Bonferroni post hoc test, or Mann-Whitney nonparametric test by using GraphPad Prism Software. Differences were considered statistically significant at P<0.05.

## Results

### Angiotensin II contributes to increased vascular TLR4 gene expression in SHRs

**Figure 1:**
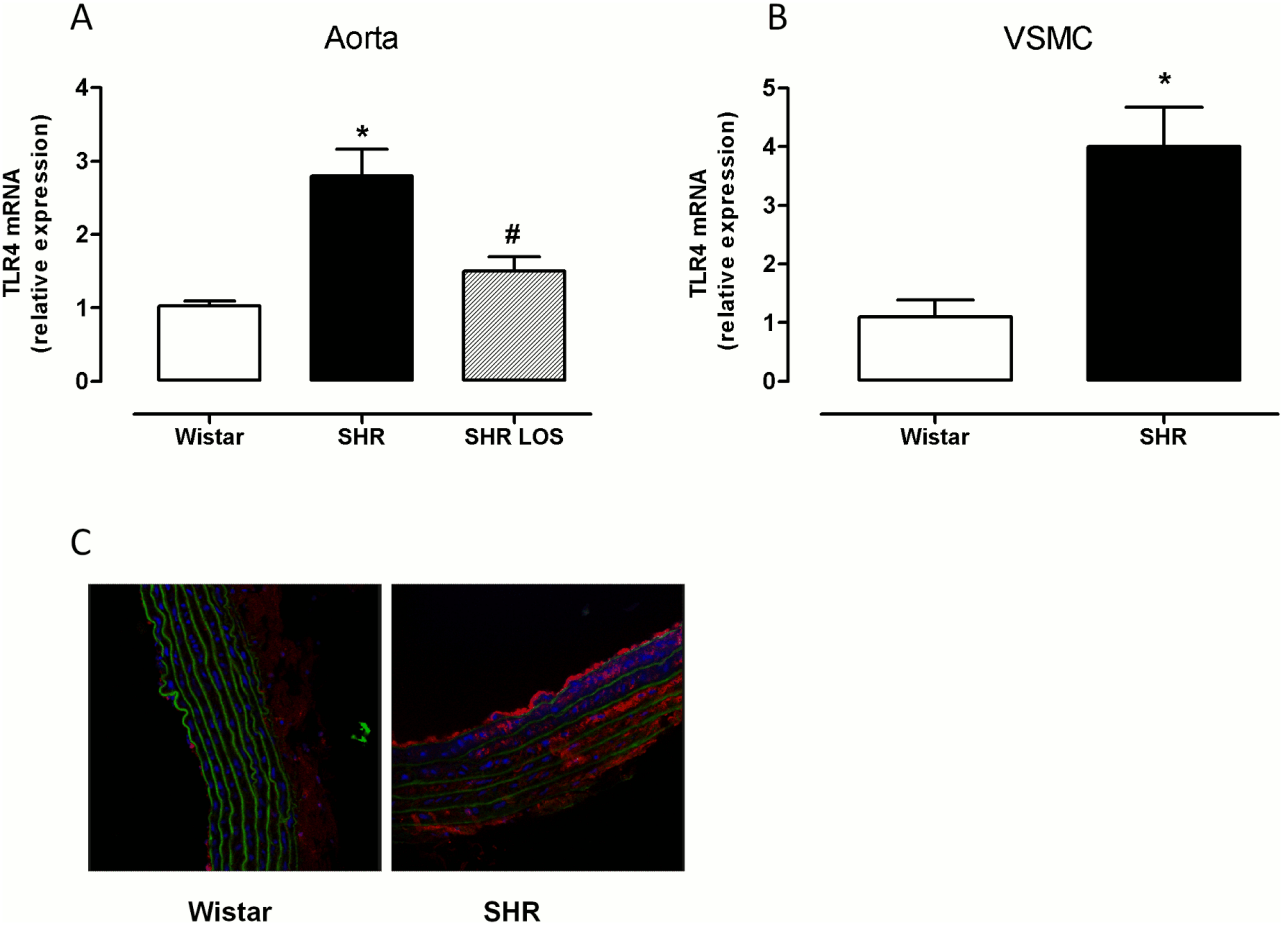
Ang II contributes to the increased TLR4 mRNA levels observed in SHRs. (A) TLR4 mRNA levels in aortic segments from Wistar, SHRs and SHRs treated with losartan (15 mg/kg·day in the drinking water, 12 weeks). (B) TLR4 mRNA levels in VSMCs from Wistar rats and SHRs. (C) Representative fluorescent confocal photomicrographs of TLR4 immunolocalization in aortic segments from Wistar and SHRs. Image size 238×238 µm. The results (mean±SEM) are expressed as the relative expression compared with the Wistar rats. *P<0.05 *vs.* Wistar, #P<0.05 *vs*. SHR using the Mann-Whitney nonparametric test. n = 6–7.

TLR4 mRNA expression was greater in aortic segments from SHRs when compared with those from Wistar rats; this greater expression was reduced after treatment of SHRs with the AT_1_ receptor antagonist losartan (**Fig. 1A**), which suggests that Ang II contributes to this increased expression. Similarly, TLR4 mRNA levels were greater in VSMCs from SHRs than from Wistar rats (**Fig. 1B**).

Immunofluorescence experiments confirm the greater expression of TLR4 in aorta from SHR when compared with Wistar rats; this receptor was localized in the three layers of the vascular wall (**Fig. 1C**).

### TLR4 inhibition reduces blood pressure and heart rate in hypertensive rats

At the end of the treatment, body weight (Wistar: 322.8±10.4 g, n = 7; SHR: 289.8±7.6 g, n = 9; anti-TLR4 SHRs: 281.5±10.7 g, n = 10) and the tibia length (Wistar: 3.8±0.1; SHR: 3.4±0.1; anti-TLR4 SHRs: 3.2±0.1 cm) was similar (P>0.05) in the three animal groups. The heart weight:tibia length ratio was greater in SHRs (0.346±0.022) as compared with Wistar rats (0.294±0.009), although the differences did not reach statistical significance (P = 0.068); treatment of SHR with anti-TLR4 antibody did not affect this ratio (0.346±0.009, P>0.05).

**Figure 2:**
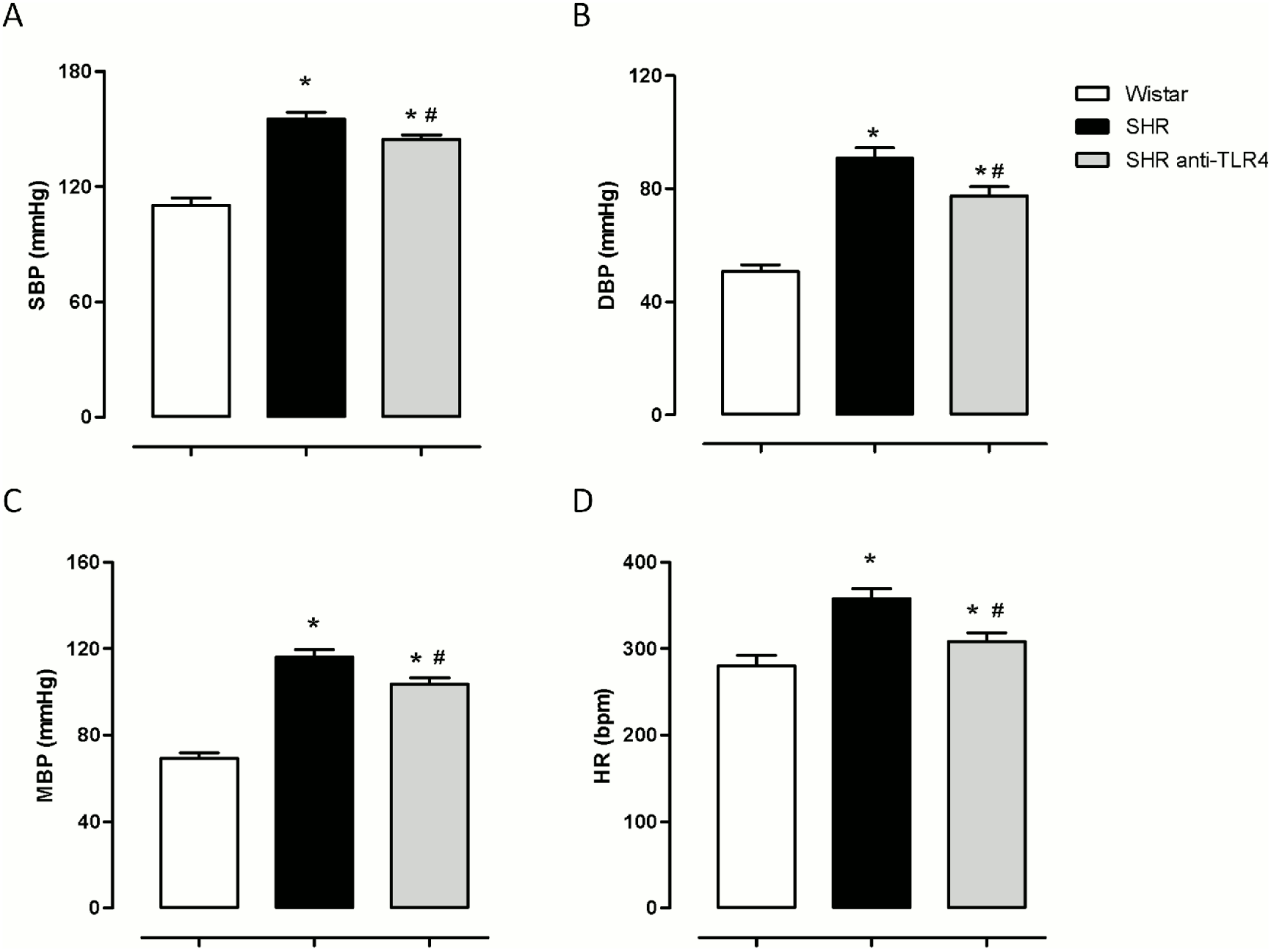
TLR4 inhibition reduces blood pressure and heart rate in hypertensive rats. (A) Systolic blood pressure (SBP), (B) diastolic blood pressure (DBP), (C) mean blood pressure (MBP) and (D) heart rate (HR) in Wistar and SHRs treated with a non-specific IgG (1 µg/day, 15 days) and SHRs treated with anti-TLR4 antibody (1 µg/day, 15 days). The results represent the mean±SEM. *P<0.05 *vs* Wistar, #P<0.05 *vs*. SHR using one way ANOVA and Bonferroni post-test. n = 8–9.

As expected, SHRs have greater levels of SBP, DBP, MBP and HR than Wistar rats (**Fig. 2A–D**). Anti-TLR4 antibody treatment of SHR lowered the SBP, DBP and MBP in SHRs (**Fig. 2A–C**) compared with the controls. Additionally, the HR also decreased with treatment (**Fig. 2D**).

### TLR4 inhibition reduces phenylephrine-elicited vasoconstriction and increases acetylcholine-elicited vasodilation in SHR aortas

**Figure 3:**
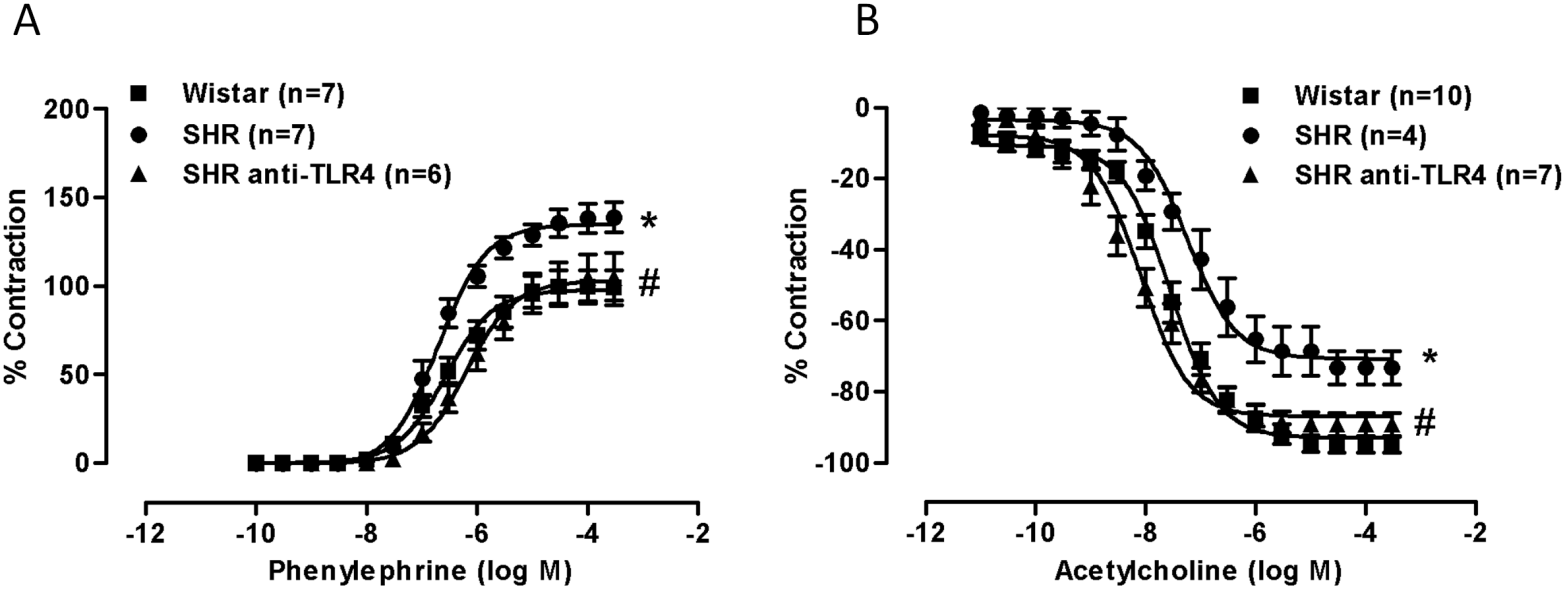
TLR4 inhibition reduces phenylephrine-elicited vasoconstriction and increases acetylcholine-elicited vasodilation in SHR aortas. Concentration-response curves to phenylephrine (A) and acetylcholine (B) in endothelium-intact aortic segments from Wistar and SHRs treated with a non-specific IgG (1 µg/day, 15 days) and SHRs treated with anti-TLR4 antibody (1 µg/day, 15 days). The results are the mean±SEM. *P<0.05 *vs* Wistar, #P<0.05 *vs*. SHR using two-way ANOVA and Bonferroni post-test. The number of animals used is shown in parentheses.

The maximum response induced by 75 mM KCl was similar in the aortic rings from both Wistar (1.87±0.01 g; n = 8) and SHRs (1.92±0.08 g, n = 9; P>0.05). Treatment of SHRs with the anti-TLR4 antibody did not modify KCl response (1.70±0.01 g, n = 10; P>0.05). The contraction induced by phenylephrine was greater in aortic segments from SHR when compared with Wistar rats (pD_2_ SHR: 6.69±0.07 *vs.* Wistar: 6.54±0.11, P>0.05; Emax SHR: 135.7±8.0% *vs.* Wistar: 100.3±9.5%, P<0.05; **Fig. 3A**); however, after anti-TLR4 antibody treatment of SHRs the phenylephrine-induced responses were lower (pD_2_ SHR anti-TLR4: 6.22±0.12, P<0.05 *vs*. SHR; Emax SHR anti-TLR4: 105.2±13.4%, P<0.05 *vs*. SHR; **Fig. 3A**). Furthermore, endothelium-dependent relaxation to acetylcholine was lower in SHRs (pD_2_ SHR: 7.25±0.19 *vs.* Wistar: 7.46±0.16, P>0.05; Emax SHR: 73.2±4.7% *vs.* Wistar: 95.3±2.4%, P<0.05; **Fig. 3B**), being these responses increased by the anti-TLR4 antibody treatment (pD_2_: 8.10±0.14, P<0.05; Emax: 89.2±3.3%, P<0.05; **Fig. 3B**).

**Figure 4:**
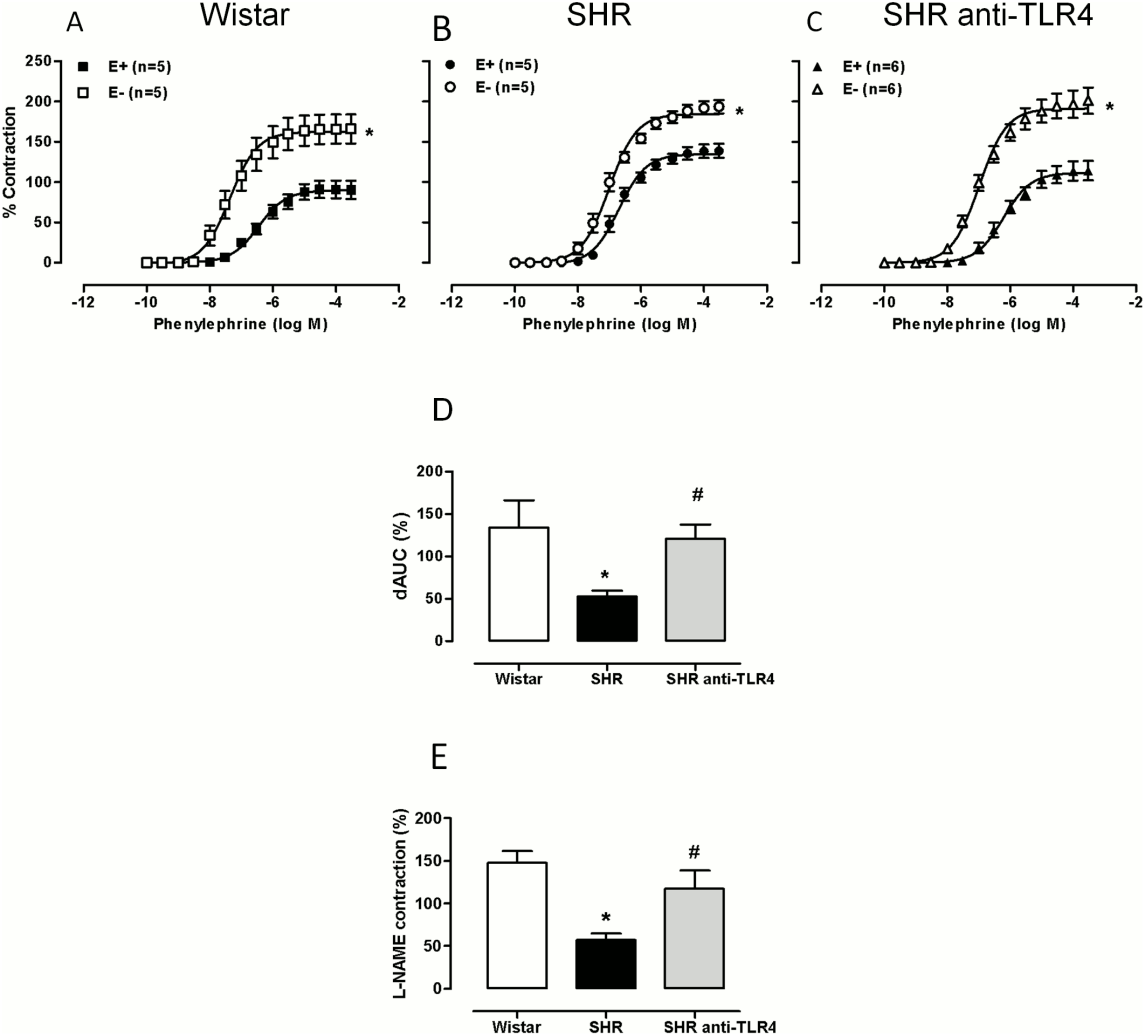
TLR4 inhibition increases endothelial modulation of vasoconstrictor responses. Concentration-response curves to phenylephrine in intact (E+) and endothelium denuded (E−) aortic segments from (A) Wistar and (B) SHRs treated with a non-specific IgG (1 µg/day, 15 days) and (C) SHRs treated with anti-TLR4 antibody (1 µg/day, 15 days). (D) Differences in the area under the concentration-response curve (dAUC) in the E– and E+ segments; AUC was calculated from the individual curve plots; the differences are expressed as a percentage of the AUC for the corresponding control situation. (E) Contractile response to L-NAME (100 µM) after phenylephrine (1 µM) contraction in segments from Wistar and SHRs treated with a non-specific IgG and SHRs treated with anti-TLR4 antibody; the results are expressed as the percentage of the previous contraction induced by phenylephrine. The results are the mean±SEM. *P<0.05 *vs.* E+ or *vs*. Wistar, #P<0.05 *vs.* SHR using two-way ANOVA and Bonferroni post-test. The number of animals used is shown in parentheses.

To evaluate the influence of endothelium in the response to phenylephrine, this layer was mechanically removed. In these conditions, the response to phenylephrine was increased in the three groups (**Fig. 4A–C**); however, this increase was smaller in SHRs, as shown by the analysis of dAUC values, while the anti-TLR4 antibody treatment restored this increase (**Fig. 4D**). These results suggest that hypertension reduces the endothelial modulation of phenylephrine responses and that the treatment reestablishes this modulation. The tension developed by the NO synthase antagonist L-NAME (100 µM) after phenylephrine contraction was lower in SHRs when compared with Wistar rats, and this contraction was increased after treatment of SHRs with the anti-TLR4 antibody (**Fig. 4E**). These results indirectly suggest that treatment significantly increases NO production. Altogether, these results allow us to propose that the anti-TLR4 antibody treatment improves endothelial dysfunction in SHRs, and this improvement most likely occurs by increasing NO bioavailability.

### TLR4 inhibition decreases the contribution of oxidative stress to the vasoconstrictor and vasodilator responses in aortas from hypertensive rats

**Figure 5:**
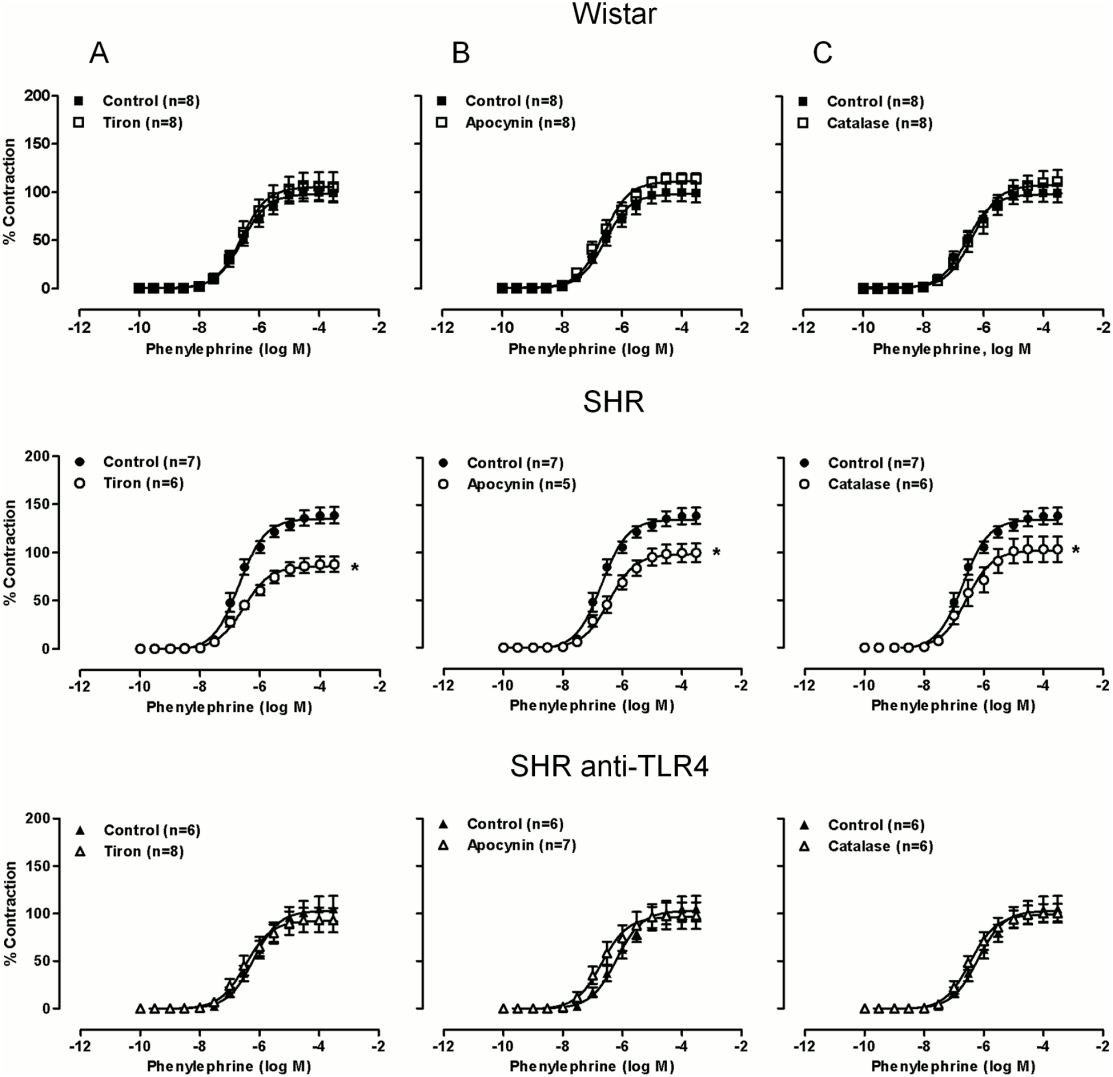
TLR4 inhibition abolishes the inhibitory effect of antioxidants on phenylephrine-induced contraction. Concentration-response curves to phenylephrine in aortic segments from Wistar and SHRs treated with a non-specific IgG (1 µg/day, 15 days) and SHRs treated with anti-TLR4 antibody (1 µg/day, 15 days) in the absence and presence of (A) tiron (1 µM), (B) apocynin (30 µM) and (C) catalase (1000 U/ml). Control curves were the same in the different situation of the three experimental groups. The results are the mean±SEM. *P<0.05 *vs.* SHR using two-way ANOVA and Bonferroni post-test. The number of animals used is shown in parentheses.

Endothelial dysfunction accompanying hypertension is frequently associated with reduction in NO bioavailability caused by increased ROS production, among other mechanisms. Therefore, we analyzed the effect of anti-TLR4 antibody treatment on the contribution of ROS to vascular function. The superoxide anion scavenger tiron, the NAD(P)H oxidase inhibitor apocynin and the hydrogen peroxide detoxificant catalase did not affect phenylephrine-induced contraction in Wistar rats, while the three drugs reduced those responses in SHRs (**Fig. 5A–C**). Treatment with the anti-TLR4 antibody completely abolished the inhibitory effect of the three antioxidants analyzed (**Fig. 5A–C**).

**Figure 6:**
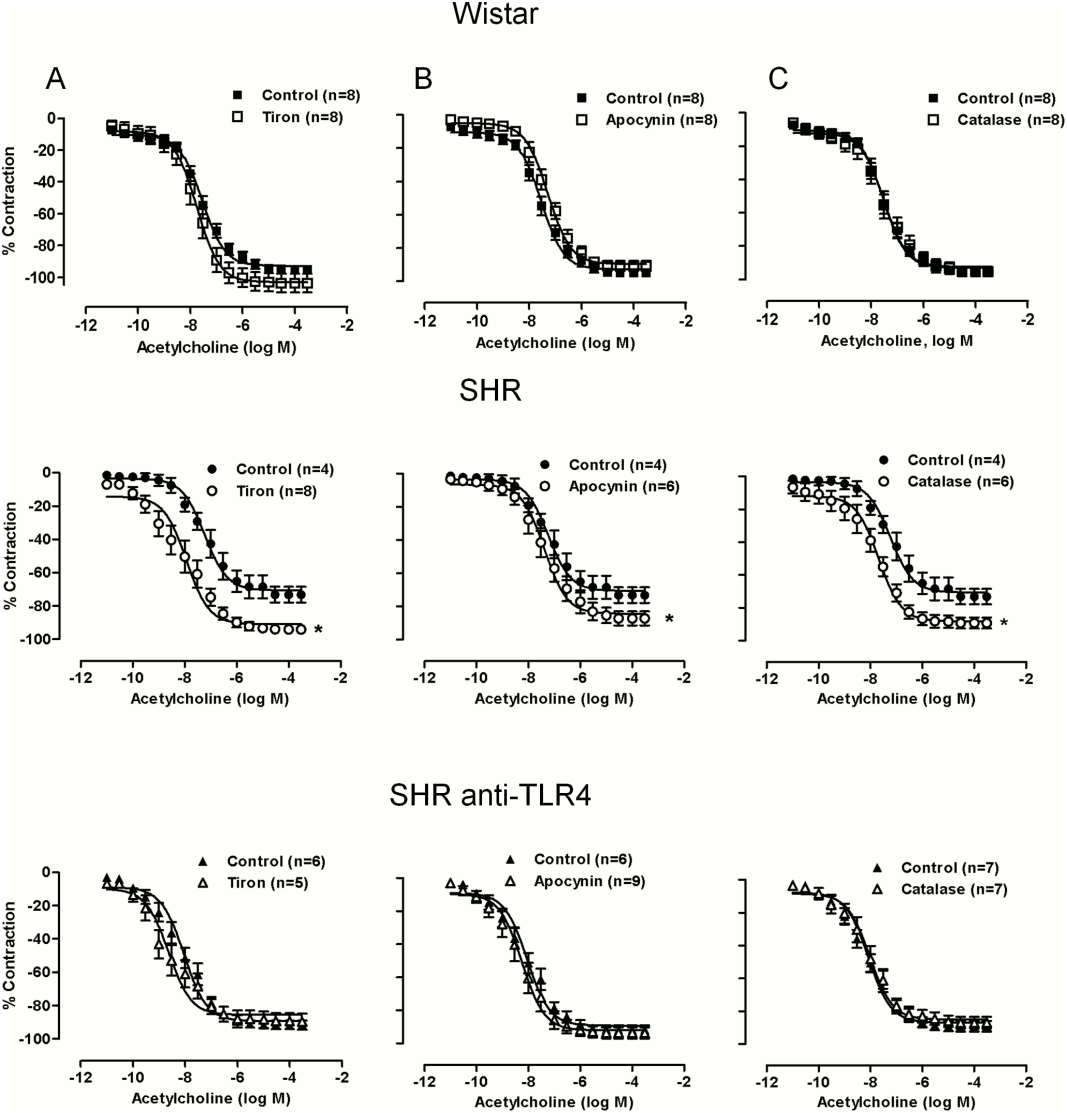
TLR4 inhibition abolishes the enhancing effect of antioxidants on acetylcholine-induced vasodilation. Concentration-response curves to acetylcholine in aortic segments Wistar and SHRs treated with a non-specific IgG (1 µg/day, 15 days) and SHRs treated with anti-TLR4 antibody (1 µg/day, 15 days) in the absence and presence of (A) tiron (1 µM), (B) apocynin (30 µM) and (C) catalase (1000 U/ml). Control curves were the same in the different situation of the three experimental groups. The results are the mean±SEM. *P<0.05 *vs.* SHR using two-way ANOVA and Bonferroni post-test. The number of animals used is shown in parentheses.

On the other hand, tiron, apocynin and catalase did not affect acetylcholine-induced relaxation in Wistar rats but increased that response in SHRs; after anti-TLR4 antibody treatment of SHRs the enhancing effect of the three compounds was abolished (**Fig. 6A–C**). Altogether, these results suggest that TLR4 increased oxidative stress and this would contribute to the impaired vascular function observed in aortas from hypertensive rats. In an attempt to investigate if these effects of TLR4 are related to the increased RAS activity in SHRs, the following experiments were carried out using cultured VSMCs stimulated with Ang II.

### Angiotensin II increases oxidative stress in VSMCs through TLR4 activation

**Figure 7:**
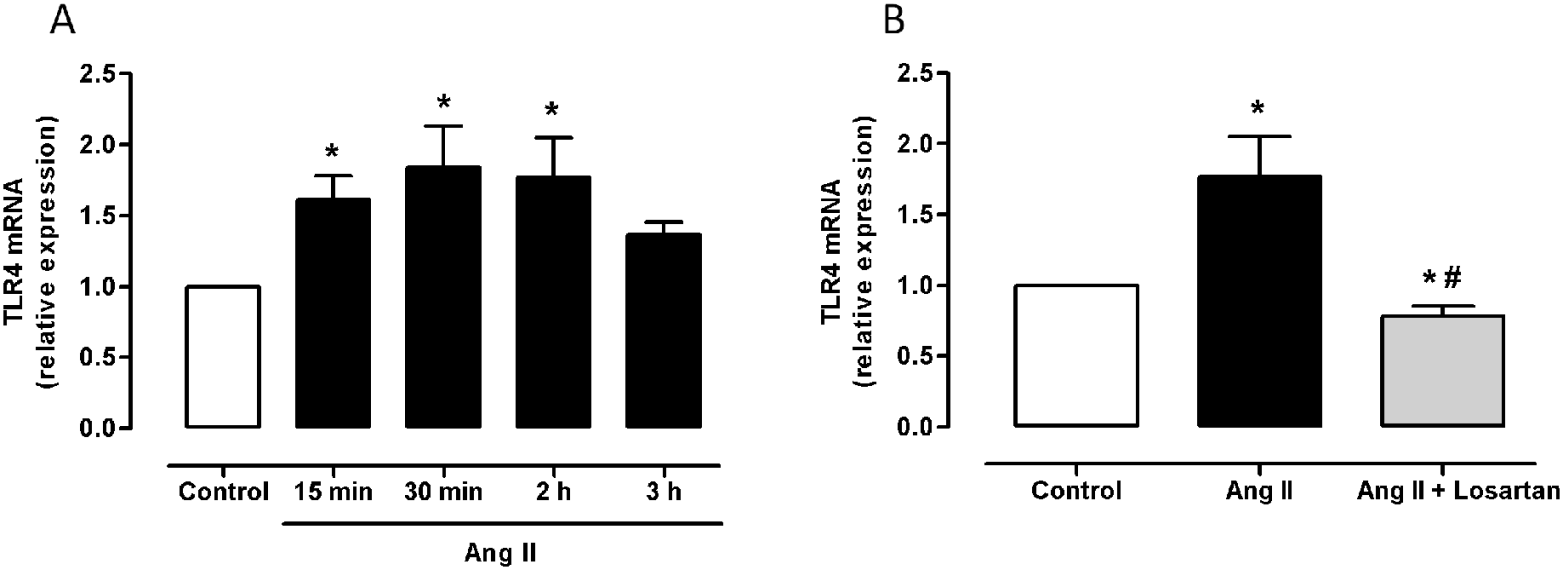
Ang II upregulates TLR4 expression in SHR VSMCs via the AT_1_ receptors. (A) Effect of Ang II (100 nM, 15 min-3 h) on TLR4 mRNA levels in SHR VSMCs. (B) Effect of Ang II (2 h) on TLR4 mRNA levels in the absence and the presence of losartan (10 µM, 1 h). The results (mean±SEM) are expressed as relative expression compared with control. *P<0.05 *vs* Control, #P<0.05 *vs* Ang II by Student’s t-test. n = 4–5.

In SHR VSMCs, Ang II (100 nM) incubation increased TLR4 mRNA levels from 15 min to 3 h (**Fig. 7A**), and this effect was reduced by preincubation with losartan (1 µM, **Fig. 7B**), which indicates the involvement of AT_1_ receptors in such induction.

**Figure 8:**
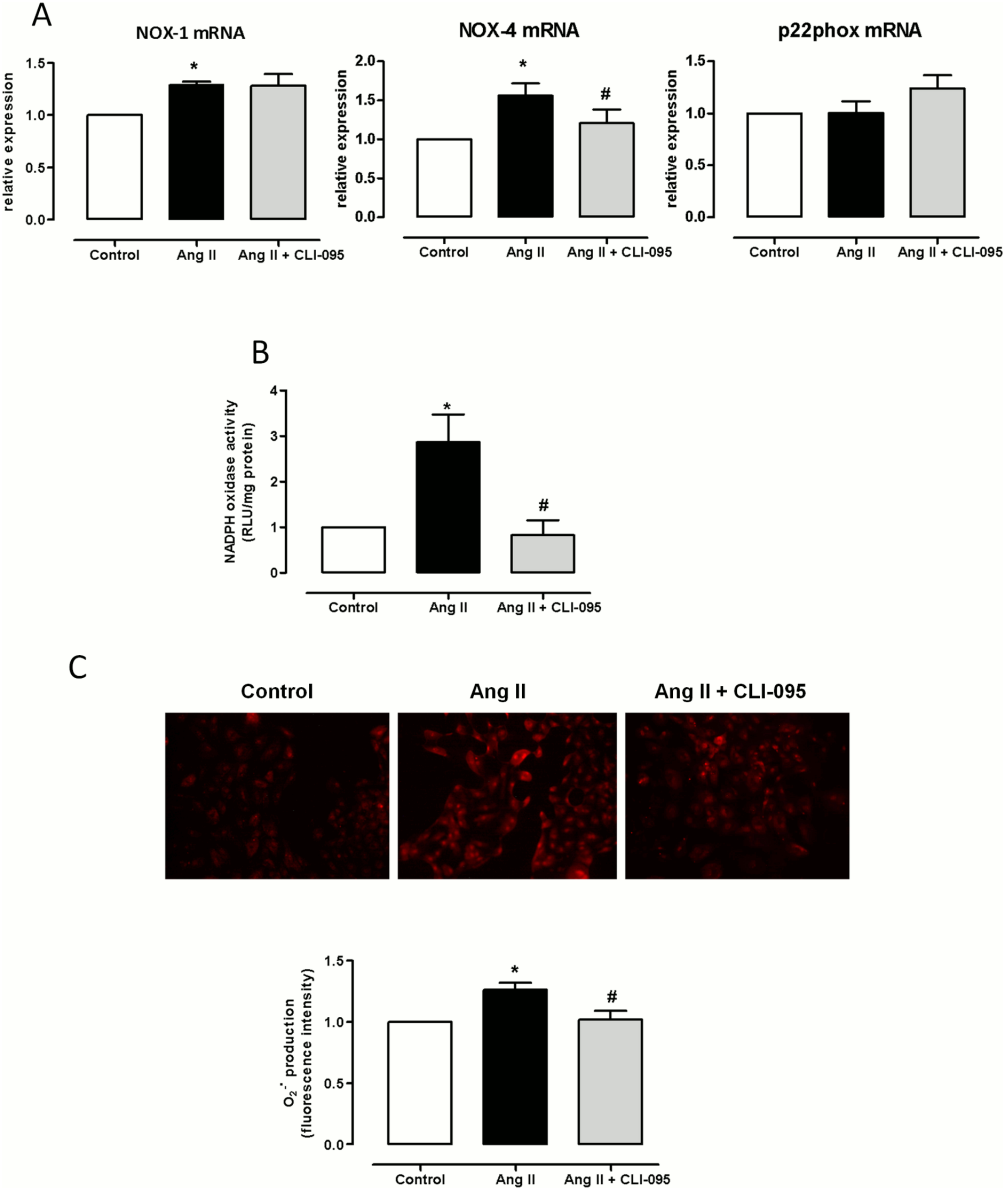
TLR4 inhibition reduces Ang II-induced NAD(P)H oxidase activity and O_2_^−^ production in VSMC from SHR. Effects of CLI-095 (1 µM, 1 h) on Ang II (100 nM, 2 h)-induced (A) NOX-1, NOX-4 and p22phox mRNA levels, (B) NAD(P)H oxidase activity and (C) O_2_^−•^ production in SHR VSMCs. The results (mean±SEM) are expressed as relative values compared with the control. *P<0.05 *vs.* Control, ^#^P<0.05 *vs.* Ang II using Student’s t-test or the Mann-Whitney nonparametric test. n = 4–6.

It has been widely shown that Ang II increases ROS production thus contributing to the inflammatory process associated with hypertension [1], [6], [36]. Accordingly, in SHR VSMCs, Ang II (100 nM, 2 h) treatment increased NOX-1 and NOX-4 mRNA levels, while p22phox mRNA levels were unaffected (**Fig. 8A**); NOX-2 mRNA levels were scarcely detected (results not shown). In addition, Ang II also increased NAD(P)H oxidase activity and the subsequent superoxide anion production (**Fig. 8B, C**). The TLR4 antagonist CLI-095 did not affect NOX-1 mRNA levels; however, CLI-095 mitigated the increased NOX-4 mRNA levels, NAD(P)H activity and superoxide anion production induced by Ang II (**Fig. 8A–C**), which indicates a role for TLR4 in the increased oxidative stress induced by Ang II.

**Figure 9:**
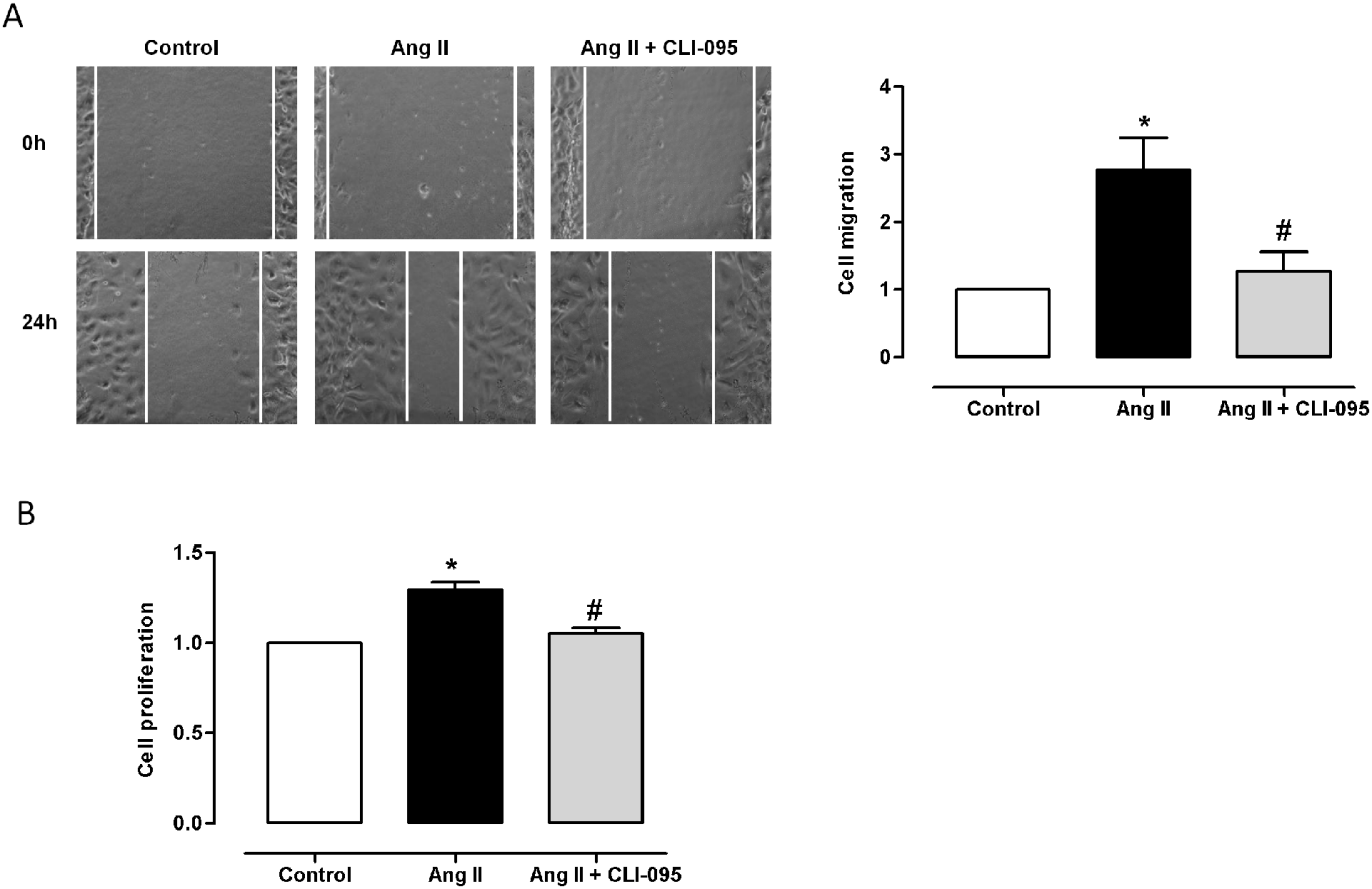
TLR4 inhibition reduces Ang II-induced cell migration and proliferation. (A) Wound healing assay showing SHR VSMC migration in response to Ang II (100 nM, 24 h) and effect of CLI-095 (1 µM, 1 h). (B) Effect of CLI-095 on Ang II-induced proliferation. The results (mean±SEM) are expressed as relative values compared with the control. *P<0.05 *vs* Control, ^#^P<0.05 *vs.* Ang II using Student’s t-test. n = 5.

The ability of Ang II to induce cell migration and proliferation is well known [37]. The TLR4 inhibitor CLI-095 also reduced the increased cell migration and proliferation induced by Ang II (100 nM, 24 h, **Fig. 9A, B**), which suggests that TLR4 contributes to these effects.

In SHR VSMCs, the TLR4 antagonist CLI-095 alone did not modify any parameter studied (results not shown).

## Discussion

The results of this study suggest that increased expression of TLR4, which is associated with increased RAS activity, contributes to the occurrence of hypertension. Additionally, this increased TLR4 expression is also involved in the previously described increased oxidative stress that most likely contributes to the endothelial dysfunction observed in hypertension.

TLR4 is expressed on the surface of different cell types including endothelial cells and VSMCs, and is involved in the recognition of and response to LPS, although many non-infectious endogenous TLR4 ligands also exist [4], [20], [21]. There is evidence regarding a role for the TLR4 signaling pathway and the innate immune response in the development of cardiovascular pathologies with an inflammatory component such as atherosclerosis [14], diabetes [26]–[28] and pre-eclampsia [29], [30]. Hypertension has also been considered as a low-grade inflammatory disease, and growing evidence shows that the immune system is involved in the pathophysiology of hypertension [2]–[4]. In the present study, we found that TLR4 expression was increased in the three layers of the vascular wall of aortic segments as well as in cultured VSMCs from SHRs compared with Wistar rats, suggesting that TLR4 from the endothelial and smooth muscle cells, and even from the adventitia, might contribute to the effects discussed below. The results are similar to that found in mesenteric resistance arteries from SHR [32] as well as in cardiomyocytes from both adults SHR and L-NAME-induced hypertensive rats [31]. Additionally, in peripheral monocytes from nondiabetic hypertensive patients, increased TLR4 gene expression has also been reported; antihypertensive treatment reduces that expression with a significantly association with the systolic and diastolic blood pressure reduction [38]. Increased TLR4 levels in SHRs can explain the enhanced vascular responses to LPS that we previously observed [7]–[9]. Furthermore, TLR4 expression seems to be associated with the development/maintenance of hypertension because treatment of SHRs with an anti-TLR4 antibody for 2 weeks reduced blood pressure, as previously described [32]. Accordingly, a recent report has shown that L-NAME failed to induce hypertension in TLR4^−/−^ mice [33]. Moreover, after the use of a neutralizing antibody *in vivo* in SHRs, the heart rate was also reduced suggesting that the cardiac effects of TLR4 can contribute to the hypertensive action of this pathway. In this sense, it has been described that activation of the TLR4 in the brainstem via AT1R contributes to the sympathoexcitation drive in heart failure [39] and recently Dange et al. [40] has shown that brain TLR4 blockade improves cardiac function in Ang II-induced hypertensive rats.

RAS contributes to the vascular alterations associated with hypertension via its proinflammatory activity in the vascular wall, including the production of ROS, cytokines and prostanoids [1], [6], [36]. Furthermore, Ang II is able to induce the inflammatory response via the TLR4 pathway [13]–[19]; in addition, AT_1_ receptor blockers (ARBs) reduce the LPS-induced innnate immune response [41]–[43] and protect against myocardial ischemia-reperfusion injury through the TLR4/NF-κB signaling pathway [44]. Accordingly, we found that Ang II increased TLR4 mRNA levels in SHR VSMCs via the AT_1_ receptors, and treatment of SHRs with losartan *in vivo* decreased the increased TLR4 levels found in this strain. These results suggest that the increased RAS activity observed in hypertension contributes to the increased TLR4 levels.

Hypertension is associated with functional vascular alterations such as endothelial dysfunction with impaired endothelium-dependent relaxations and increased vasoconstrictor responses. Endothelial dysfunction is a prognostic factor for cardiovascular events in patients with essential hypertension [45]. Our results demonstrate that the anti-TLR4 antibody treatment improved the vasodilator responses to acetylcholine in SHRs. Additionally, the anti-TLR4 antibody reduced vasoconstrictor responses to phenylephrine, in agreement with results obtained in mesenteric resistance arteries [32]. Moreover, the increased phenylephrine response after endothelial denudation was greater in anti-TLR4 antibody-treated SHRs. Altogether, this study suggests for the first time, to the best of our knowledge, that TLR4 contributes to the endothelial dysfunction observed in hypertension. In keeping with this, our group and others have previously shown that LPS administration induces endothelial dysfunction in both peripheral [46], [47] and cerebral arteries [9]. Additionally, Liang et al. [48] reported that the *in vivo* and *in vitro* administration of LPS causes endothelial dysfunction in the arteries of wild-type mice, but not those of TLR4-mutated mice. The proposed role of TLR4 in endothelial dysfunction and the above mentioned increased TLR4 expression found in hypertensive animals can explain the greater impairment of bradykinin-induced relaxation elicited by LPS that was observed in the middle cerebral arteries of hypertensive rats [9].

One of the mechanisms that explain the endothelial dysfunction induced by TLR4 activation is the reduction of NO contribution to vascular responses. Accordingly, anti-TLR4 treatment improved endothelium-dependent relaxation, which in aorta is dependent on NO [49]; in addition, the L-NAME-induced contraction was greater in SHR aortic segments after anti-TLR4 antibody treatment, suggesting that this treatment might increase NO bioavailability. Augmented oxidative stress is one of the most generally accepted mechanism to explain the reduced NO bioavailability in hypertension [50], [51]. There is evidence indicating that upon TLR4 activation, LPS increases the generation of ROS, such as O_2_^−^ and H_2_O_2_, from NAD(P)H oxidase, and these ROS are involved in NF-κB activation and the subsequent expression of proinflammatory cytokines [22]–[25]. In the present study we found that the effect of antioxidants (apocynin, catalase and tiron) on both vascular contraction and relaxation disappeared after anti-TLR4 antibody treatment, suggesting that TLR4 contributes to the increased ROS production and endothelial dysfunction observed in hypertension. Accordingly, in obesity- and diabetes-associated endothelial dysfunction by increasing oxidative stress TLR4 plays a key role [48]. Recently, it was proposed that the greater ROS production caused by DAMPs release in L-NAME-induced hypertensive mice contributes to the vascular alterations found in this model [33]. TLR4 could also contribute to endothelial dysfunction by reducing NO production. Indeed, in cardiac microvascular endothelial cells, a reduction of eNOS expression and NO production via TLR4 signaling has been described under hypoxia/reoxygenation conditions [52]. However, we cannot discard COX-dependent mechanisms associated with TLR4 activation that might contribute to the vascular dysfunction associated with hypertension [32].

RAS plays an important role in increasing the oxidative stress present in hypertension [1], [5], [6], [12], [36]. In SHR VSMCs, the TLR4 antagonist mitigated the increased NOX-4 expression, NAD(P)H oxidase activity and superoxide anion production induced by Ang II. These results support the contribution of RAS-induced TLR4 in the oxidative stress observed in hypertension. Some authors have also described the role of TLR4 in the Ang II effects mediated by ROS production. Thus, after Ang II release, osteocalcin activates PKC/TLR4/ROS/COX-2, which mediates the transformation of fibroblasts to myofibroblasts [19]. Additionally, TLR4/MyD88-mediated oxidative stress is involved in Ang II-induced mesangial cell apoptosis [17].

Ang II induces VSMC proliferation and migration that contribute to the progression of many vascular diseases, including hypertension [37]. Yuen et al. [19] have shown that the Ang II-induced migration of rat adventitial fibroblasts is mediated by TLR4 activation. Additionally, TLR4 contributes to the increased proliferation and migration induced by other stimuli [53], [54]. Accordingly, the increased cell proliferation and migration induced by Ang II was reversed by TLR4 antagonists, thus suggesting that increased TLR4 expression is functionally associated with structural alterations that can also contribute to hypertension.

In conclusion, this study demonstrates, for the first time, that the increased RAS activity observed in hypertension stimulates the TLR4 pathway, contributing to the occurrence of hypertension. Additionally, by inducing oxidative stress, TLR4 leads to the endothelial dysfunction that is characteristic of this pathology. In recent years, several studies had demonstrated the role of adaptive immunity in the pathogenesis of hypertension [2], [3]. Our results also suggest the impact of the TLR4 signaling pathway on the development of this inflammatory pathology and its associated vascular alterations. However, further investigations that deeply analyze the role of immunity in hypertension and end-organ damage will help develop therapies for this global disease.

## References

[1] Savoia C, Schiffrin EL (2007) Vascular inflammation in hypertension and diabetes: molecular mechanisms and therapeutic interventions. Clin Sci 112: 375–384. 17324119 10.1042/CS20060247

[2] Schiffrin EL (2013) The immune system: role in hypertension. Can J Cardiol 29: 543–548. 22902155 10.1016/j.cjca.2012.06.009

[3] Schiffrin EL (2014) Immune mechanisms in hypertension and vascular injury. Clin Sci 126: 267–274. 24144355 10.1042/CS20130407

[4] McCarthy CG, Goulopoulou S, Wenceslau CF, Spitler K, Matsumoto T, et al (2014) Toll-like receptors and damage-associated molecular patterns: novel links between inflammation and hypertension. Am J Physiol Heart Circ Physiol 306: H184–H196. 24163075 10.1152/ajpheart.00328.2013 PMC3920129

[5] Alvarez Y, Pérez-Girón JV, Hernanz R, Briones AM, García-Redondo A, et al (2007) Losartan reduces the increased participation of cyclooxygenase-2-derived products in vascular responses of hypertensive rats. J Pharmacol Exp Ther 321: 381–388. 17244722 10.1124/jpet.106.115287

[6] Touyz RM, Briones AM (2011) Reactive oxygen species and vascular biology: implications in human hypertension. Hypertens Res 34: 5–14. 20981034 10.1038/hr.2010.201

[7] Briones AM, Alonso MJ, Marín J, Salaices M (1999) Role of iNOS in the vasodilator responses induced by L-arginine in the middle cerebral artery from normotensive and hypertensive rats. Br J Pharmacol 126: 111–120. 10051127 10.1038/sj.bjp.0702281 PMC1565787

[8] Briones AM, Alonso MJ, Marín J, Balfagón G, Salaices M (2000) Influence of hypertension on nitric oxide synthase expression and vascular effects of lipopolysaccharide in rat mesenteric arteries. Br J Pharmacol 131: 185–194. 10991910 10.1038/sj.bjp.0703552 PMC1572313

[9] Hernanz R, Briones AM, Alonso MJ, Vila E, Salaices M (2004) Hypertension alters role of iNOS, COX-2, and oxidative stress in bradykinin relaxation impairment after LPS in rat cerebral arteries. Am J Physiol Heart Circ Physiol 287: H225–H234. 15001439 10.1152/ajpheart.00548.2003

[10] Bhagat K, Vallance P (1997) Inflammatory cytokines impair endothelium-dependent dilatation in human veins in vivo. Circulation 96: 3042–3047. 9386173 10.1161/01.cir.96.9.3042

[11] García-Redondo AB, Briones AM, Beltrán AE, Alonso MJ, Simonsen U, et al (2009) Hypertension increases contractile responses to hydrogen peroxide in resistance arteries through increased thromboxane A_2_, Ca^2+^, and superoxide anion levels. J Pharmacol Exp Ther 328: 19–27. 18818375 10.1124/jpet.108.144295

[12] Martínez-Revelles S, Avendaño MS, García-Redondo AB, Alvarez Y, Aguado A, et al (2013) Reciprocal relationship between reactive oxygen species and cyclooxygenase-2 and vascular dysfunction in hypertension. Antioxid Redox Signal 18: 51–65. 22671943 10.1089/ars.2011.4335

[13] Wolf G, Bohlender J, Bondeva T, Roger T, Thaiss F, et al (2006) Angiotensin II upregulates toll-like receptor 4 on mesangial cells. J Am Soc Nephrol 17: 1585–1593. 16675600 10.1681/ASN.2005070699

[14] Otsui K, Inoue N, Kobayashi S, Shiraki R, Honjo T, et al (2007) Enhanced expression of TLR4 in smooth muscle cells in human atherosclerotic coronary arteries. Heart Vessels 22: 416–422. 18044001 10.1007/s00380-007-1001-1

[15] Ji Y, Liu J, Wang Z, Liu N (2009) Angiotensin II induces inflammatory response partly via toll-like receptor 4-dependent signaling pathway in vascular smooth muscle cells. Cell Physiol Biochem 23: 265–276. 19471094 10.1159/000218173

[16] Ji Y, Liu J, Wang Z, Liu N, Gou W (2009) PPARγ agonist, rosiglitazone, regulates angiotensin II-induced vascular inflammation through the TLR4-dependent signaling pathway. Lab Invest 89: 887–902. 19451898 10.1038/labinvest.2009.45

[17] Lv J, Jia R, Yang D, Zhu J, Ding G (2009) Candesartan attenuates Angiotensin II-induced mesangial cell apoptosis via TLR4/MyD88 pathway. Biochem Biophys Res Commun 380: 81–86. 19161983 10.1016/j.bbrc.2009.01.035

[18] Wu J, Yang X, Zhang YF, Zhou SF, Zhang R, et al (2009) Angiotensin II upregulates Toll-like receptor 4 and enhances lipopolysaccharide-induced CD40 expression in rat peritoneal mesothelial cells. Inflamm Res 58: 473–482. 19271152 10.1007/s00011-009-0012-z

[19] Yuen CY, Wong SL, Lau CW, Tsang SY, Xu A, et al (2012) From skeleton to cytoskeleton: osteocalcin transforms vascular fibroblasts to myofibroblasts via angiotensin II and Toll-like receptor 4. Circ Res 111: e55–66. 22679141 10.1161/CIRCRESAHA.112.271361

[20] Tsan MF, Gao B (2004) Endogenous ligands of Toll-like receptors. J Leukoc Biol 76: 514–519. 15178705 10.1189/jlb.0304127

[21] Kawai T, Akira S (2010) The role of pattern-recognition receptors in innate immunity: update on Toll-like receptors. Nat Immunol 11: 373–384. 20404851 10.1038/ni.1863

[22] Asehnoune K, Strassheim D, Mitra S, Kim JY, Abraham E (2004) Involvement of reactive oxygen species in Toll-like receptor 4-dependent activation of NF-κB. J Immunol 172: 2522–2529. 14764725 10.4049/jimmunol.172.4.2522

[23] Ryan KA, Smith MF Jr, Sanders MK, Ernst PB (2004) Reactive oxygen and nitrogen species differentially regulate Toll-like receptor 4-mediated activation of NF-κB and interleukin-8 expression. Infect Immun 72: 2123–2130. 15039334 10.1128/IAI.72.4.2123-2130.2004 PMC375203

[24] Park HS, Chun JN, Jung HY, Choi C, Bae YS (2006) Role of NADPH oxidase 4 in lipopolysaccharide-induced proinflammatory responses by human aortic endothelial cells. Cardiovasc Res 72: 447–455. 17064675 10.1016/j.cardiores.2006.09.012

[25] Pacquelet S, Johson JL, Ellis BA, Brzeinska AA, Lane WS, et al (2007) Croos-talk between IRAK-4 and the NADPH oxidase. Biochem J 403: 451–461. 17217339 10.1042/BJ20061184 PMC1876389

[26] Devaraj S, Dasu MR, Rockwood J, Winter W, Griffen SC, et al (2008) Increased Toll-like Receptor (TLR) 2 and TLR4 expression in monocytes from patients with Type 1 Diabetes: further evidence of a proinflammatory state. J Clin Endocrinol Metab 93: 578–583. 18029454 10.1210/jc.2007-2185 PMC2243229

[27] Dasu MR, Devaraj S, Park S, Jialal I (2010) Increased toll-like receptor (TLR) activation and TLR ligands in recently diagnosed type 2 diabetic subjects. Diabetes Care 33: 861–868. 20067962 10.2337/dc09-1799 PMC2845042

[28] Xie BG, Jin S, Zhu WJ (2014) Expression of toll-like receptor 4 in maternal monocytes of patients with gestational diabetes mellitus. Exp Ther Med 7: 236–240. 24348797 10.3892/etm.2013.1360 PMC3861517

[29] Kim YM, Romero R, Oh SY, Kim CJ, Kilburn BA, et al (2005) Toll-like receptor 4: a potential link between “danger signals,” the innate immune system, and preeclampsia? Am J Obstet Gynecol 193: 921–927. 16157088 10.1016/j.ajog.2005.07.076

[30] Pineda A, Verdin-Terán SL, Camacho A, Moreno-Fierros L (2011) Expression of Toll-like Receptor TLR-2, TLR-3, TLR-4 and TLR-9 is increased in placentas from patients with preeclampsia. Arch Med Res 42: 382–391. 21843566 10.1016/j.arcmed.2011.08.003

[31] Eiβler R, Schmaderer C, Rusai K, Kühne L, Sollinger D, et al (2011) Hypertension augments cardiac Toll-like receptor 4 expression and activity. Hypertens Res 34: 551–558. 21248757 10.1038/hr.2010.270

[32] Bomfim GF, Dos Santos RA, Oliveira MA, Giachini FR, Akamine EH, et al (2012) Toll-like receptor 4 contributes to blood pressure regulation and vascular contraction in spontaneously hypertensive rats. Clin Sci 122: 535–543. 22233532 10.1042/CS20110523 PMC4004345

[33] Sollinger D, Eiβler R, Lorenz S, Strand S, Chmielewski S, et al (2014) Damage-associated molecular pattern activated Toll-like receptor 4 signalling modulates blood pressure in L-NAME-induced hypertension. Cardiovasc Res 101: 464–472. 24302630 10.1093/cvr/cvt265

[34] Xavier FE, Davel AP, Rossoni LV, Vassallo DV (2003) Time-dependent hyperreactivity to phenylephrine in aorta from untreated diabetic rats: role of prostanoids and calcium mobilization. Vascul Pharmacol 40: 67–76. 12646412 10.1016/s1537-1891(02)00315-4

[35] Martín A, Pérez-Girón JV, Hernanz R, Palacios R, Briones AM, et al (2012) Peroxisome proliferator-activated receptor-γ activation reduces cyclooxygenase-2 expression in vascular smooth muscle cells from hypertensive rats by interfering with oxidative stress. J Hypertens 30: 315–326. 22179086 10.1097/HJH.0b013e32834f043b

[36] Virdis A, Duranti E, Taddei S (2011) Oxidative Stress and Vascular Damage in Hypertension: Role of Angiotensin II. Int J Hypertens 2011: 916310. 21747985 10.4061/2011/916310 PMC3124711

[37] Mehta PK, Griendling KK (2007) Angiotensin II cell signaling: physiological and pathological effects in the cardiovascular system. Am J Physiol Cell Physiol 292: C82–C97. 16870827 10.1152/ajpcell.00287.2006

[38] Marketou ME, Kontaraki JE, Zacharis EA, Kochiadakis GE, Giaouzaki A, et al (2012) TLR2 and TLR4 gene expression in peripheral monocytes in nondiabetic hypertensive patients: the effect of intensive blood pressure-lowering. J Clin Hypertens (Greenwich) 14: 330–335. 22533660 10.1111/j.1751-7176.2012.00620.x PMC8108838

[39] Ogawa K, Hirooka Y, Kishi T, Sunagawa K (2011) Brain AT_1_ receptor activates the sympathetic nervous system through toll-like receptor 4 in mice with heart failure. J Cardiovasc Pharmacol 58: 543–549. 21822148 10.1097/FJC.0b013e31822e6b40

[40] Dange RB, Agarwal D, Masson GS, Vila J, Wilson B, et al (2014) Central blockade of TLR4 improves cardiac function and attenuates myocardial inflammation in Angiotensin II-induced hypertension. Cardiovasc Res 103: 17–27. 24667851 10.1093/cvr/cvu067

[41] Sánchez-Lemus E, Benicky J, Pavel J, Larrayoz IM, Zhou J, et al (2009) Angiotensin II AT_1_ blockade reduces the lipopolysaccharide-induced innate immune response in rat spleen. Am J Physiol Regul Integr Comp Physiol 296: R1376–R1384. 19225144 10.1152/ajpregu.90962.2008 PMC2689834

[42] Sanchez-Lemus E, Murakami Y, Larrayoz-Roldan IM, Moughamian AJ, Pavel J, et al (2008) Angiotensin II AT_1_ receptor blockade decreases lipopolysaccharide-induced inflammation in the rat adrenal gland. Endocrinology 149: 5177–5188. 18556352 10.1210/en.2008-0242 PMC2582913

[43] Dasu MR, Riosvelasco AC, Jialal I (2009) Candesartan inhibits Toll-like receptor expression and activity both in vitro and in vivo. Atherosclerosis 202: 76–83. 18495130 10.1016/j.atherosclerosis.2008.04.010 PMC2676176

[44] Yang J, Jiang H, Yang J, Ding J-W, Chen L-H, et al (2009) Valsartan preconditioning protects against myocardial ischemia-reperfision injury through TLR4/NFκB signalling pathway. Mol Cell Biochem 330: 39–46. 19370315 10.1007/s11010-009-0098-1

[45] Perticone F, Ceravolo R, Pujia A, Ventura G, Iacopino S, et al (2001) Prognostic significance of endothelial dysfunction in hypertensive patients. Circulation 104: 191–196. 11447085 10.1161/01.cir.104.2.191

[46] Brandes RP, Koddenberg G, Gwinner W, Kim DY, Kruse HJ, et al (1999) Role of increased production of superoxide anions by NAD(P)H oxidase and xanthine oxidase in prolonged endotoxemia. Hypertension 33: 1243–1249. 10334819 10.1161/01.hyp.33.5.1243

[47] Pleiner J, Mittermayer F, Schaller G, MacAllister RJ, Wolzt M (2002) High doses of vitamin C reverse Escherichia coli endotoxin-induced hyporeactivity to acetylcholine in the human forearm. Circulation 106: 1460–1464. 12234948 10.1161/01.cir.0000030184.70207.ff

[48] Liang CF, Liu JT, Wang Y, Xu A, Vanhoutte PM (2013) Toll-Like receptor 4 mutation protects obese mice against endothelial dysfunction by decreasing NADPH Oxidase isoforms 1 and 4. Arterioscler Thromb Vasc Biol 33: 777–784. 23413427 10.1161/ATVBAHA.112.301087

[49] Freitas MR, Schott C, Corriu C, Sassard J, Stoclet JC, et al (2003) Heterogeneity of endothelium-dependent vasorelaxation in conductance and resistance arteries from Lyon normotensive and hypertensive rats. J Hypertens 21: 1505–1512. 12872044 10.1097/00004872-200308000-00014

[50] Cai H, Harrison DG (2000) Endothelial dysfunction in cardiovascular diseases: the role of oxidant stress. Circ Res 87: 840–844. 11073878 10.1161/01.res.87.10.840

[51] Schulz E, Gori T, Münzel T (2011) Oxidative stress and endothelial dysfunction in hypertension. Hypertens Res 34: 665–673. 21512515 10.1038/hr.2011.39

[52] Zhang Z, Li W, Sun D, Zhao L, Zhang R, et al (2011) Toll-like receptor 4 signaling in dysfunction of cardiac microvascular endothelial cells under hypoxia/reoxygenation. Inflamm Res 60: 37–45. 20652722 10.1007/s00011-010-0232-2

[53] de Graaf R, Kloppenburg G, Kitslaar PJ, Bruggeman CA, Stassen F (2006) Human heat shock protein 60 stimulates vascular smooth muscle cell proliferation through Toll-like receptors 2 and 4. Microbes Infect 8: 1859–1865. 16843693 10.1016/j.micinf.2006.02.024

[54] Pi Y, Zhang LL, Li BH, Guo L, Cao XJ, et al (2013) Inhibition of reactive oxygen species generation attenuates TLR4-mediated proinflammatory and proliferative phenotype of vascular smooth muscle cells. Lab Invest 93: 880–887. 23774581 10.1038/labinvest.2013.79

